# Supplementary material for: Nasopharyngeal swabs vs. saliva sampling for SARS-CoV-2 detection: A cross-sectional survey of acceptability for caregivers and children after experiencing both methods
Source: PLoS One. 2022 Jul 8;17(7):e0270929. doi: 10.1371/journal.pone.0270929 (PMC9269879; doi:10.1371/journal.pone.0270929)
Supplement: S1 File — (DOCX) [file pone.0270929.s002.docx]

**Supporting information - Survey**

SECTION A. The following questions are to be answered by the **patient (the study participant)**. The primary decision maker should read the following instructions to the child before they answer the questions:

Script: These faces show how much something can hurt. The first face on the left shows no pain. The faces show more and more pain as they move to the right. The last face on the right shows very much pain.

1. Select the face that shows how much pain you had during the SWAB IN THE NOSE.

[FPS-R shown]

2. Select the face that shows how much pain you had during the SPIT TEST.

[FPS-R shown]

3. If you had to have another test for COVID-19, which type of test would you choose? (circle your answer)

SWAB IN THE NOSE SPIT TEST NO PREFERENCE

SECTION B. The following questions are to be answered by the **primary decision maker** present. If no **primary decision maker** is present, then the study participant can answer if they are 12 years old or older.

4. How many times has the study participant had a COVID test since the beginning of the pandemic (including today)?

1 2 3 4 5 or more

5. If you are the you are the primary decision maker, how many times have you been tested for COVID?

0 1 2 3 4 5 or more Not applicable (I am the study participant)

6. Consider the following situation: the study participant is required to get tested for COVID in order to return to school. The only available test is the SWAB IN THE NOSE.

How likely are you to have the participant tested?

Very unlikely Unlikely Neutral Likely Very likely

7. Consider the following situation: the study participant is required to get tested for COVID in order to return to school. The only available test is the SPIT TEST.

How likely are you to have the participant tested?

Very unlikely Unlikely Neutral Likely Very likely

8. Consider the following situation: the study participant has a fever and a cough. A health care professional recommends that the study participant gets tested for COVID. The only available test is the SWAB IN THE NOSE.

How likely are you to have the participant tested?

Very unlikely Unlikely Neutral Likely Very likely

9. Consider the following situation: the study participant has a fever and a cough. A health care professional recommends that the study participant get tested for COVID. The only available test is the SPIT TEST.

How likely are you to have the participant tested?

Very unlikely Unlikely Neutral Likely Very likely

10. Consider the following situation: the study participant has been in close contact with someone infected with COVID. The study participant does not have any symptoms. The only available test is the SWAB IN THE NOSE.

How likely are you to have the participant tested?

Very unlikely Unlikely Neutral Likely Very likely

11. Consider the following situation: the study participant has been in close contact with someone infected with COVID. The study participant does not have any symptoms. The only available test is the SPIT TEST.

How likely are you to have the participant tested?

Very unlikely Unlikely Neutral Likely Very likely

12.The SPIT TEST may not be as accurate as the SWAB IN THE NOSE. Suppose the spit test would miss 1 out of 10 COVID infections detectable by the SWAB IN THE NOSE. If the participant needed testing, which would you choose?

SWAB IN THE NOSE SPIT TEST No preference

13. Consider the following situation: a numbing spray could be put in the nose of the study participant before the SWAB IN THE NOSE to decrease pain. You would have to wait 10 minutes for the spray to have an effect before doing the test.

How likely are you to want the numbing spray for the study participant?

Very unlikely Unlikely Neutral Likely Very likely

14. What is the highest level of education you have completed?

Less than High School High School CEGEP College University – Undergraduate -University - Graduate University - Post Graduate Trade School Professional Degree (ex. Law school, Medical School)

15. Please leave any additional comments below: ________________________________________________________________________________________________________________________________________________________________________________________________________________________________________________________________________________________________________________________
