# Supplementary material for: Nasopharyngeal swabs vs. saliva sampling for SARS-CoV-2 detection: A cross-sectional survey of acceptability for caregivers and children after experiencing both methods
Source: PLoS One. 2022 Jul 8;17(7):e0270929. doi: 10.1371/journal.pone.0270929 (PMC9269879; doi:10.1371/journal.pone.0270929)
Supplement: Attachment — Submitted filename: Reviewers Response.docx [file pone.0270929.s003.docx]

May 9^th,^ 2022

Dr. A. M. Abd El-Aty

Academic Editor, PLOS ONE

Dear Dr. Abd El-Aty,

Please find enclosed the revised manuscript entitled “Nasopharyngeal Swabs vs. Saliva-based Sampling for SARS-CoV-2 Detection: A Cross-Sectional Survey of Acceptability for Caregivers and Children after Experiencing Both Methods” which we are resubmitting to PLOS ONE for consideration of publication as an original investigation.

We thank the PLOS ONE editors and reviewers for their thoughtful and insightful review. The constructive feedback provided by the reviewers and the editorial board have resulted in a strengthened manuscript. We have addressed each comment on a point-by-point basis below. We appreciate the opportunity to have our revised paper reconsidered for publication. With our region currently in an official 6th wave of COVID-19 and the effect of future SARS-CoV-2 virus variants still uncertain, we believe our paper to be of continued relevance and interest.

In summary, in this revised version we have conducted an additional exploratory analysis based on the saliva testing method used, we reviewed the literature, updated our references and adapted the manuscript to the current state of the pandemic. We also modified our methods to include additional details pertaining to the survey itself.

We look forward to your review of our revised manuscript, and we would be pleased to answer any questions you may have regarding the study.

Sincerely,

François Gagnon MD FRCPC	Stuart Harman MD FRCPC

Pediatric Emergency Medicine Fellow	Division of Emergency Medicine

Children’s Hospital of Eastern Ontario	Department of Pediatrics

University of Ottawa	Children’s Hospital of Eastern Ontario

Assistant Professor, University of Ottawa

**COMMENTS OF REVIEWER #1:**

**1) The authors present a very relevant study, one which can be informative for future diagnostic practices. We have seen first-hand through the pandemic how testing aversion can lead to delayed testing and testing avoidance, both which can have serious consequences for public health. We must learn from our experiences through the pandemic and continue to innovate the rest of our clinical diagnostics to improve acceptability to the patient to encourage greater adherence to testing - and less fear of clinical space. While a few questionnaires have been issued to evaluate people's thoughts and experiences with sampling technique, this is the first that I am aware of to also study the physical response to these samples. Thus, I commend the authors on their study and bringing this issue to light.**

We thank the reviewer for their positive comments. We share the feeling that obtaining information about the patient experience is relevant, especially since COVID-19 may remain a public health concern over the next few years. Non-invasive testing is likely to remain an important tool.

**Major comments:**

**2) Lines 74-76: There was a recent study evaluating test acceptability in schools which may require this text to be updated a little: https://journals.sagepub.com/doi/full/10.1177/00333549221074395. The authors might like to review this recent publication in case any of the references need to be updated or if this paper adds any additional support to their work: https://www.dovepress.com/acceptability-of-community-saliva-testing-in-controlling-the-covid-19--peer-reviewed-fulltext-article-PPA**

Response:

Thank you for informing us of this new and pertinent literature. We modified the introduction to acknowledge the relevance of the first paper you mentioned while arguing that our study maintains its pertinence. The following paragraph was added:

**Introduction (lines 79 to 83):**

*“In a recent study, school-age children, parents and school personnel were offered COVID-19 surveillance via a saliva sampling method. They were then asked whether they would have consented to testing if a nasal swab was offered instead. Of those who accepted the saliva-based method, 36% would have refused a nasal swab.(6) Of note, more than half of the students in the study had never actually experienced a nasopharyngeal swab when answering the question.”*

**3)** **Methods: the sampling procedure for both NP and saliva sampling is missing. This is important to the study for the reader to understand especially, which saliva sampling technique they received and how they found this. It would also be helpful to attach the saliva sampling SOP/IFU into the appendix so people can evaluate this - ie, were people coaxed? This can help with collection. The authors could also discuss that 1 mL is a relatively large amount to collect considering many major saliva tests only require 50-300 ul of sample, meaning that less could be collected, further improving acceptability.**

We apologize for the lack of transparency relating to the method of saliva sampling. This is an especially important point since we are evaluating the patient perspective with regards to the different sampling methods utilized. We opted to include descriptions of the collection techniques directly in the methods. Children that were asked to produce sputum were encouraged to do so by the nurse. We have also addressed in the discussion that the amount of saliva collected was quite large and may have had an effect on the acceptability of the test. We believe these changes have strengthened the manuscript.

**Materials and Methods (Lines 116 to 126):**

*“Children and caregivers presenting to the regional COVID-19 test centre who consented to the parent study were offered participation in the present sub-study. As part of the parent study, participants underwent both a nasopharyngeal swab and a saliva sample. Nasopharyngeal swabs were inserted into the nasaopharynx and gently rotated for 5 to 10 seconds. Saliva samples were collected via 2 different methods based on age. Children 6 to 8 years old had their saliva tested by a sponge-based kit. A small sponge was inserted between their cheek and teeth for 1 minute. Children 9 years and older were asked and encouraged to expectorate 1ml of saliva in a tube. Once both samples were collected by nursing staff, a team member (FG, SH) administered a 15-item questionnaire to the children and their primary decision makers (for medical decisions). Adolescents (12 years old and older) could identify as being their own primary medical decision makers. “*

**Discussion** **(Lines 215 to 224):**

*“Unlike saliva ~~SB~~ sampling, however, the acceptability of nasopharyngeal swabs was different when the indication for testing changed. Primary decision makers were less accepting of future nasopharyngeal swabs when their child was an asymptomatic contact of ~~with~~ a COVID-positive case, compared to when it was a requirement for returning to school. The acceptability of the saliva ~~SB~~ sample, in contrast, remained high across all scenarios. Participants who preferred a nasopharyngeal swab commented that producing 1ml of saliva was a slow and unpleasant process. It should be noted that most saliva-based assays require only 0.05 to 0.3ml for testing.(5) Alternatively, the sponge collection process appeared to have been well tolerated. After experiencing both tests, the vast majority of children indicated a preference for saliva sampling, which was rated, almost unanimously, as painless.”*

**4) Conclusion: why is there a sudden mention of Mers?**

This was an oversight. Thank you for identifying the mistake. The text now reads as follows:

**Conclusion (Lines 295 to 296)**

*“Further study of SARS-CoV-2 ~~Mers-CoV-2~~ sampling accuracy and acceptability will help determine the ideal application for each modality.”*

**Minor comments:**

**5) As 'NP' and 'nasopharyngeal' contribute the same word count and as 'saliva-based' and 'SB' also contribute the same word count (or in many places could simply be 'saliva'), I recommend that 'saliva' and 'nasopharyngeal' be written in full. As this work will have broad appeal, this will be easier for the less-experienced reader to follow along with.**

Thank you for the suggestion. We have made the modifications as per your recommendation throughout the manuscript. We are hopeful that it improved the readability of the manuscript.

**6)** **Update 'SARs-CoV-2' to 'SARS-CoV-2' throughout.**

Thank you for noticing these mistakes. We have updated the manuscript and SARS-CoV-2 now appears throughout.

**7) Line 225: This makes a good reference for how it is the robustness of saliva testing (and sample collection) that can affect sensitivity (rather than straight 'saliva' itself) - this is important as with robust methods, saliva performs equal to or better than swabs: https://www.thelancet.com/journals/lanres/article/PIIS2213-2600%2821%2900178-8/fulltext**

Thank you for bringing this important reference to our attention. This finding is particularly relevant since acceptability is correlated to test performance. We hope to have improved the manuscript with the following modification.

**8)** **Discussion (Lines 252 to 257):**

*“While initial meta-analyses have estimated sensitivity of saliva samples to be similar to nasopharyngeal swabs,(3, 4), ~~a subsequent study by~~ Mestdagh et al.(14) found that the ~~te~~ sensitivity varied widely according to saliva collection technique and viral load. ~~Using a similar outpatient setting and testing indications as for our study, they found sensitivity in SB sampling to be as low as 21.9% and as high as 93.9% using the NP swab result as the gold standard.~~ Tan et al.(15) also found that test performance varied significantly according to saliva collection and processing technique, and suggested in their review that saliva assays may perform better than nasopharyngeal swabs in optimal conditions.”*

**COMMENTS OF REVIEWER #2:**

**Majors Comments:**

**9) Why were there two different saliva sampling methods used in the study? As this is evaluating preference the NP stays constant, but a sponge collection and spit would add additional variables to the already limited number of patients. Ideally additional participants would be added to remove the sponge method. If not possible, the data should be separated between method types.**

Thank you for the comment. The protocol of the parent study utilized 2 different sampling methods because children under the age of 8 have a hard time producing sputum for collection. A sponge method was used in the younger age group. We nevertheless included children 6 to 8 years old into our survey because we believed their experience to be pertinent and important as well. It is true that additional participants would have been ideal, but unfortunately it was not possible.

We agree that having 2 different sampling methods adds a variable, but we argue the following: 1) Since almost all children stated having no significant pain or distress irrespective of the saliva collection method, we believe that grouping together all children irrespective of the saliva testing method to be reasonable. 2) Dividing our small sample size would have a deleterious effect on power. 3) As recommended, a post-hoc exploratory sub-group analysis to identify significant difference between both saliva collection methods is necessary. We are thankful for the remarks above which prompted us to investigate further.

We obtained the following results:

Demographics:

Similar participants characteristics, apart from age (which determined the saliva testing method). See the table below:

Main outcome:

Results regarding the likelihood of accepting saliva testing were similar between both saliva testing methods.

A mention could be made that perhaps children who underwent the sponge test were less likely to agree to nasopharyngeal testing compared to those who were completing the spit test. We believe that this finding is more likely related to the younger age of those tested with a sponge than the saliva testing method (confounder).

-Secondary outcomes:

Average saliva testing pain: Sponge method 0/10, spit method 0/10. There is no difference between both groups.

Preferred testing method: Sponge group – 100% preferred the saliva method.. Spit group – 88% preferred the saliva method. We believe this finding to be interesting and have added that finding to the result section.

Trade-off (supposing 10% inferior sensitivity for saliva test): Spit group: 67% preferred nasopharyngeal swabs, sponge group: 63% preferred nasopharyngeal swabs. There is no clinically significant difference between both groups.

Desire for a numbing spray: Spit group 50% would want a numbing spray, sponge group 63% would want a numbing spray. There is no clinically significant difference between both groups.

In summary, we propose the following modifications to the article:

**Materials and Methods (Lines 156 to 158)**

*“We conducted an exploratory sub-group analysis on age (<12 vs. >=12 years) to better understand the effect of age on the various outcomes. We also carried out an exploratory post-hoc analysis on the effect of the saliva testing method (expectoration vs sponge) on outcomes.”*

**Main results (Lines 204 to 205)**

*“Across all scenarios, the likelihood of accepting a saliva test did not appear to be influenced by the type of saliva testing method used (sponge vs. expectoration).”*

**Main results (Lines 197 to 200)**

*“The two participants who preferred the nasopharyngeal swab stated that it was quicker than the saliva test (expectoration) or that the process of expectorating 1ml of saliva was tedious. All participants (N=8) who underwent a sponge technique for their saliva collection, preferred the saliva test to the nasopharyngeal swab.”*

**Discussion (Lines 216 to 223)**

“Primary decision makers were less accepting of future nasopharyngeal swabs when their child was an asymptomatic contact of a COVID-positive case, compared to when it was a requirement for returning to school. The acceptability of the *saliva* ~~SB~~ sample, in contrast, remained high across all scenarios. *Participants who preferred a nasopharyngeal swab commented that producing 1ml of saliva was a slow and unpleasant process. It should be noted that most saliva-based assays require only 0.05 to 0.3ml for testing.(5) Alternatively, the sponge collection process appeared to have been well tolerated.*”

**10)** **I would suggest adding more details about the questions used in the survey. At the moment the material and methods of what was asked was unclear and requires going to supplemental data to review the survey to understand what was asked.**

Thank you for the suggestion. We have added more details to the questions to improve the reading experience.

**Methods (Lines 130 to 142)**

*“Three hypothetical clinical scenarios were developed to assess acceptability of both tests in different contexts: 1) The study participant requires testing in order to return to school; 2) The study participant has fever and cough and testing is recommended by a health care professional; 3) The study participant is a close contact of someone with COVID-19, but remains asymptomatic. Primary decision makers rated their willingness to accept a test on a 5-point Likert scale (e.g., very unlikely to very likely; (see Supporting Information). Procedural pain was assessed using the validated Faces Pain Scale-Revised (FPS-R).(9) Participants’ preference ~~for testing~~ for sampling modality was assessed by asking them to indicate which they would choose if a future test was required, and the desire for an intranasal numbing agent was assessed with a yes or no question ~~direct binary questions~~. ~~In the final hypothetical scenario the primary decision maker had to consider a potential trade-off of test comfort versus test sensitivity.~~ Additionally, primary decision makers were asked to consider a potential trade-off of test comfort versus test sensitivity by indicating their test preference in a hypothetical scenario where saliva sampling was 10% less sensitive than a nasopharyngeal swab.”*

**Minor Comments:**

**11) Ln 257 and 261: This seems to be a typo as its stated as Mers and not SARS.**

We apologize for the mistake. The text now reads: “SARS-CoV-2” on both lines.

**One complaint and issue with saliva testing is obtaining enough volume to perform all testing. Was there any issues to obtain the 1mL from subjects?**

Thank you for raising this important point. All subjects completed the collection adequately. However, one participant who stated preferring the nasopharyngeal swab to the saliva test mentioned that the collection process was tedious. It was also noted by another reviewer that the quantity of saliva obtained (1ml) was significantly more than what is required in other protocols. We have addressed these points in the following way.

**Methods (Lines 117 to 122):**

*“As part of the parent study, participants underwent both a nasopharyngeal swab and a saliva sample. Nasopharyngeal swabs were inserted into the nasaopharynx and gently rotated for 5 to 10 seconds. Saliva samples were collected via 2 different methods based on age. Children 6 to 8 years old had their saliva tested by a sponge-based kit. A small sponge was inserted between their cheek and teeth for 1 minute. Children 9 years and older were asked and encouraged to expectorate 1ml of saliva in a tube.”*

**Results (Lines 165 to 168)**

*“Forty-eight eligible patients were included in the analysis with a median age of 10 years (IQR: 9, 13). Clinical characteristics are presented in Table 1. All participants enrolled in the study were able to complete both sampling methods and answer the survey.”*

**Discussion (Lines 218 to 224):**

*“The acceptability of the saliva sample, in contrast, remained high across all scenarios. One of the two participants who preferred a nasopharyngeal swab commented that producing 1ml of saliva was a slow and unpleasant process. It should be noted that most saliva-based assays require only 0.05 to 0.3ml for testing.(5) Alternatively, the sponge collection process appeared to have been well tolerated. After experiencing both tests, the vast majority of children indicated a preference for saliva sampling, which was rated, almost unanimously, as painless.”*

**I would add in the limitations that the majority of primary decision makers had some college education or higher, which may have skewed results, especially when asking about sensitivity.**

Thank you for your observation. We added the following comment in the limitations section:

**Limitations (lines 282 to 285)**

*“The education level of the recruited families was higher than what might be expected of the general population and could have an impact on the generalizability of the results. This is possibly due to the urban location of the testing center.”*

**COMMENTS OF REVIEWER #3:**

**Major comments:**

**This was a very nice study looking at the acceptability of saliva and NP swab for testing children for COVID-19. This is particularly interesting because almost all other studies have focused solely on measures of test sensitivity and accuracy as opposed to patient comfort and ease. While NP swabs are the gold-standard, they are onerous to perform and so it is good to know when the slight increase in sensitivity for the test might outweigh concerns about the patient anxiety and comfort, particularly children. I thought the paper was quite good overall and suggest just some additional discussion/background to help readers place the results in context of other studies on COVID-19 testing:**

We thank you for your thoughtful comments. We agree that the patient experience is an important aspect, especially since the impact of the disease and its management has been so widespread and prolonged.

**11) I think it might be helpful to include some discussion of limitations of both techniques in general. For instance some patients cannot do a saliva test because they cannot self collect a sample (e.g., very young infants, patients with certain disabilities). Some patients are very difficult to get NP swabs on because of nasal anatomical/pathological issues. There's also typically a difference in overall cost of the tests with saliva typically being a bit cheaper overall since saliva tests do not require specialized swabs/supplies or highly trained medical staff to collect.**

Thank you for the comment. We added some information in the introduction relating to the limitations of the nasopharyngeal swabs and saliva sampling. We also added a comment regarding the potential advantages of saliva testing you have suggested. This appears in the text as:

**Introduction (Lines 70 to 78)**

“ *Saliva sampling potentially results in less discomfort and risk of viral transmission during testing, decreased utilization of personnel protective equipment, and reduced cost compared to nasopharyngeal swabs.(5, 6) Additionally, nasopharyngeal swabs are contra-indicated in some circumstances (eg. nasal anatomic pathology, coagulopathy) and have been associated with rare complications (eg. retained swabs, epistaxis, cerebrospinal fluid leakage).(7) Saliva sampling by expectoration may not be possible for all patients, neonates for example, but saliva can also be sampled with an oral sponge.* While pediatric data is under-represented in this literature, the presumption has been that saliva sampling would be particularly preferable for children.”

**12)** **The authors also do not discuss other less invasive swabs (anterior nasal, oral, etc). There have been some studies using these in other settings where NP swabs are not easy to administer.**

Thank you, we have taken your suggestion and added a comment on how our work may relate to other forms of less invasive sampling:

**Discussion** **(Lines 271 to 275)**

*“In addition to saliva collection and nasopharyngeal swabs, other alternatives examined in the literature include anterior nasal swabs, throat swabs and combinations of both. It is likely that similar factors identified in this research influence the acceptability of other testing modalities. Additional research is required to further define the acceptability of emerging SARS-CoV-2 testing techniques.(5)”*

**13)** **One of the more interesting findings is that parents would still be ok using saliva over NP swabs even if the NP swab was 10% more accurate. This might be a good place to discuss the relative accuracy of these two testing modalities.**

Thank you for your comment. It is indeed interesting that caregivers are split on the issue (66% prefer the nasopharyngeal swab). In the Discussion section we acknowledge that 10% was a hypothetical number chosen to assess caregiver willingness to trade some level of comfort for accuracy, and we have elaborated on what was in our original discussion to include an additional supporting reference. The text now appears as:

**Discussion (Lines 248 to 257)**

*“In our study we asked participants if they would accept a 10% reduction in sensitivity using saliva sampling instead of nasopharyngeal swabbing, to determine whether primary decision makers would be willing to trade a reduction in test accuracy for sampling comfort. The actual difference in sensitivity between saliva sampling and nasopharyngeal swabs is not definitively known at present, and likely varies in different clinical scenarios. While initial meta-analyses have estimated sensitivity of saliva samples to be similar to nasopharyngeal swabs,(3, 4), ~~a subsequent study by~~ Mestdagh et al.(14) found that the ~~te~~ sensitivity varied widely according to saliva collection technique and viral load. ~~Using a similar outpatient setting and testing indications as for our study, they found sensitivity in SB sampling to be as low as 21.9% and as high as 93.9% using the NP swab result as the gold standard.~~ Tan et al.(15) also found that test performance varied significantly according to saliva collection and processing technique, and suggested in their review that saliva assays may perform better than nasopharyngeal swabs in optimal conditions.”*

**14) This was done on a population of patients that had symptoms or known exposures to SARS-CoV-2 and thus, one would assume that they would be more likely to want better diagnostics than they would if this test was for a screening application. It may be useful to have a follow up survey of healthcare professionals or reference some work in the literature that surveys clinicians to compare the acceptability of saliva over NP swabbing in different applications. I would assume that clinicians would in general prefer NP swabbing for diagnostics as it is the gold standard but the data on pain and patient acceptability may sway their decision based on the reason for the COVID-19 test.**

This is a very interesting thought. It would be a great to know to what degree clinicians balance test comfort and patient acceptability when determining provider acceptability for saliva sampling vs. NP swabs. We have searched through the literature and found no work on the topic, one of the main issues being that it is not clear that NP swabs outperform saliva testing. Surveying clinicians on testing recommendations may be an interesting future research topic.

**15) Overall, this was a well designed study, which fills a clear gap in our understanding of the COVID-19 testing landscape.**

We are thankful for your appreciation of our work, as well as the efforts you have put into improving the manuscript.

**COMMENTS OF REVIEWER #4:**

**Major comments:**

**16) This is a very relevant, well-conceived and well-written study of patient preferences in the context of a rapid increase in the experience of upper respiratory sampling in all age groups. I listened to my asymptomatic niece scream during a required NP swab for a dental appointment, with an absolutely massive swab, and wondered why we couldn’t have SB screening for kids like in BC?? How individuals weigh the risks and benefits in the context of sampling quality is a fascinating element of this study – that more pain is acceptable with more gain, and whether the case is symptomatic, asymptomatic, and/or required to test might all influence which test is preferable. I am very intrigued by the older kids who preferred NP, such an interesting perspective.**

Thank you very much for your complimentary comments. Thank you as well for the valuable constructive feedback in the following comments, which we have taken to heart in enhancing our manuscript.

 **17) Your opening statement in the introduction seems no longer quite accurate post-Omicron. Not suggesting any specific changes, but it would be nice to see some additional perspectives in Intro/Discussion on how your findings might be different now, when the “scenario” is declining availability of free, trackable testing and rampant pauci-symptomatic infections at school?**

We agree that while COVID-19 testing is still an important and ongoing component of the effort to manage disease, the opening line of the introduction likely overstated it’s current role in pandemic control. We have changed the word “central” to the adjective “important”, so the line now reads:

**Introduction (Lines 66 to 67):**

*“Identification and isolation of individuals infected with SARS-CoV-2 are important components of the pandemic control effort.”*

Additionally, we thank you for the suggestion of acknowledging that test acceptability may evolve as the approach to COVID-19 testing and management changes over time. To this end, we have expanded and modified a paragraph of the Discussion to incorporate this idea, and to recognize the Omicron variant of SARS-CoV-2 (this includes an additional reference as well):

**Discussion (Lines 258 to 270):**

*“Determining the ideal test to either screen for, or diagnose, COVID-19 is an ongoing area of research that must take into account the circumstances and goals of testing. With the onset of the Omicron variant, which seems to be more transmissible but potentially less severe than previous SARS-CoV-2 variants, testing policies continue to adapt. This will also be true if future variants arise with different clinical characteristics.(16) ~~Test accuracy, cost, feasibility and acceptance are all important factors which can change with patient population and geography; these factors must be understood to create effective testing strategies.~~ Test accuracy, cost, feasibility and acceptance are all important factors which must be understood to pivot into effective testing strategies. Our study contributes insight into test acceptability for the pediatric population after children have experienced different sampling methods in a community setting. The implication that acceptability varies by testing rationale more readily when the sampling method is uncomfortable will remain relevant over the evolving course of the pandemic, particularly as the utility and perceived value of testing changes over time. Future studies should further elucidate how different populations respond to the experience of COVID-19 testing in other settings, such as the inpatient setting or when patients are highly symptomatic.”*

**18) How did you put the survey on the electronic device and link to RedCap, in detail? Software? Scripts? Device used? This is just for my information because I want to do this next month!**

Thank-you for your interest in this! We have indeed found that REDCap is an ideal tool for research data collection, and our institution has a REDCap administrator. Our ED team also has a REDCap assistant. With their help we are able to setup REDCap access through a web browser on portable tablets (in this case specifically, iPads). We would be happy to offer any support we can with this to reviewer #4, and for a detailed conversation on this topic specifically it would be best to email author Stuart Harman ([sharman@cheo.on.ca](mailto:sharman@cheo.on.ca))

**19)** **I am curious about differences in swabs used for children (thickness, fluffiness vs. sharpness of swab head) and how painful each type might be, for example, if an “adult-sized” swab was used on a little one – could this account for some of the difference between yours and Moisset’s study?**

This is a very interesting consideration. The swabs used in children are the same ones used in adults, in this case a nylon flocked swab of 2 mm thickness at the tip. At this time, there are no special swabs designed for nasopharyngeal sampling specifically in children. This may well be a contributing factor to the degree of pain reported by children, although our study was not designed to properly compare the difference in pain experienced by children vs. adults. As Moisset’s study did not enrol any children, there are a number of uncontrolled factors which could explain differences in pain between our group and theirs. This highlights a potential area for future study. The point that we aim to express in our discussion is that despite the pain reported by children with Nasopharyngeal swabs, their caregivers were mostly willing to accept the procedure again if indicated in the future.

**20) Figure 1: Add y axis title (“% of participants”), remove “%” from tick labels, y axis should end at 100, not go past it. In legend, “Saliva test” should be “Saliva-based test” or just use “SB” and “NP” to be consistent with text. Figure Caption: “at least likely” is ambiguous, do you mean just the top two Likert points? Always use curly quotation marks.**

We have reformatted the y axis as suggested, removing the “%” symbol from the labels and adding “% of Participants” to the Label. The y axis also now ends at 100. To make the legend most consistent with the manuscript text, we have changed the label “Saliva-based test” to “Saliva Sample”. Additionally, as the text already informs the reader that “To facilitate data analysis, answers to the 5-point Likert were dichotomized to likely and unlikely (including neutral answers)”, we have modified the figure caption for clarity. The updated figure and caption now appear as:

**Main Results (Lines 184 - 185)**

**Figure 1.** Percentage of participants responding they would be likely to want COVID-19 testing, based on the sampling method and scenario proposed.

**21) Figure 2: Add y axis title (“% of participants”), remove “%” from tick labels, only label every second tick (at 10% rather than 5% intervals). Figure caption is unclear, how about: “Self-reported pain after NP swab using revised Face Pain Scale (FPS-Re, percent of participants).”**

We have reformatted the y axis as suggested, removing the “%” symbol from the labels and adding “% of Participants” to the Label. The y axis is also now labelled at 10% instead of 5% intervals. We have taken the suggestion for modifying the figure caption. The revised figure and caption now appear as:

**Main Results (Lines 209 to 210)**

**Figure 2.** Self-reported pain after NP swab using revised Face Pain Scale (FPS-R, Percent of Participants)

**22) Figure 3: In legend, “Saliva test” should be “Saliva-based test” to be consistent with text. Figure caption is unclear, how about: “Preferred test in case of future need for testing (percent of participants).”**

The legend has been modified so that “Saliva test” is now “Saliva sample”, as this is most consistent with the current manuscript text. We have also taken the suggestion for modification of the figure caption. The figure and caption now appear as:

**Main Results (Line 211)**

**Figure 3**. Preferred test in case of future need for testing (Percent of Participants)

**23)** **Is “Interpretation-Limitations-Conclusion” a suggested PLoS One structure? Just “Discussion” (no sub-heads) seems preferable.**

“Interpretation-Limitations-Conclusion” is not a specific style mandated by the the PLoS One submission guidelines. We have altered the heading of the “Interpretation” section to now read “Discussion”. Stylistically, we would still prefer to leave the sub-heading of “Limitations” and the heading of “Conclusions”, as we believe in this case that such labels will benefit the reader. We will, of course, readily defer to the preferences of the editor. In addition, we have altered the sub-headings in the “Methods” section (now titled “Materials and Methods”), so as to limit the number of sub-headings to 3 and respect the PLoS One manuscript format. The sub-heading “Selection of Participants” no longer appears, though the text is retained in full within the “Study Design and Settings” portion of the “Materials and Methods” section. Likewise, the sub-heading “Ethics Approval” has been removed while the statement on ethics approval remains in full. We have also removed the sub-heading “Survey Tool”, while retaining the full text explanation of the survey tool under the “Study Procedure” sub-heading.

**24)** **This paper is low on refs. It is a unique study, so this makes sense, however some additional context about saliva testing for COVID and other infections, and pediatric screening vs. diagnostics in general, would be much appreciated.**

We have found that the reviewer is correct in suggesting there is a paucity of relevant literature on saliva testing in children. In the course of addressing this and other reviewer comments, we have added several statements and references related to saliva sampling. In particular, these appear in the manuscript as listed below.

**Introduction (Lines 70 to 78)**

“Saliva sampling potentially results in less discomfort and risk of viral transmission during testing, decreased utilization of personnel protective equipment, and reduced cost compared to nasopharyngeal swabs.(5,6) Additionally, nasopharyngeal swabs are contra-indicated in some circumstances (eg. nasal anatomic pathology, coagulopathy) and have been associated with rare complications (eg. retained swabs, epistaxis, cerebrospinal fluid leakage).(7) Saliva sampling by expectoration may not be possible for all patients, neonates for example, but saliva can also be sampled with an oral sponge. While pediatric data is under-represented in this literature, the presumption has been that saliva sampling would be particularly preferable for children.

**Introduction (Lines 79 to 83)**

*“In a recent study, school-age children, parents and school personnel were offered COVID-19 surveillance via a saliva sampling method. They were then asked whether they would have consented to testing if a nasal swab was offered instead. Of those who accepted the saliva-based method, 36% would have refused a nasal swab.(6) Of note, more than half of the students in the study had never actually experienced a nasopharyngeal swab when answering the question."*

**Discussion (Lines 258 to 262)**

*“Determining the ideal test to either screen for, or diagnose, COVID-19 is an ongoing area of research that must take into account the circumstances and goals of testing. With the onset of the Omicron variant, which seems to be more transmissible but potentially less severe than previous SARS-CoV-2 variants, testing policies continue to adapt. This will also be true if future variants arise with different clinical characteristics.(16)”*

*“In addition to saliva collection and nasopharyngeal swabs, other alternatives examined in the literature include anterior nasal swabs, throat swabs and combinations of both. It is likely that similar factors identified in this research influence the acceptability of other testing modalities. Additional research is required to further define the acceptability of emerging SARS-CoV-2 testing techniques.(5)"*

**References added:**

Tan SH, Allicock O, Armstrong-Hough M, Wyllie AL. Saliva as a gold-standard sample for SARS-CoV-2 detection. Lancet Respir Med. 2021;9(6):562-4.

Kim DH, Kim D, Moon JW, Chae SW, Rhyu IJ. Complications of Nasopharyngeal Swabs and Safe Procedures for COVID-19 Testing Based on Anatomical Knowledge. J Korean Med Sci. 2022;37(11):e88.

McLaughlin HP, Worrell MC, Malone S, Dawson P, Maricque B, Halpin JL, et al. Acceptance of Saliva-Based Specimen Collection for SARS-CoV-2 Testing Among K-12 Students, Teachers, and Staff. Public Health Rep. 2022:333549221074395.

Menni C, Valdes AM, Polidori L, Antonelli M, Penamakuri S, Nogal A, Louca P, May A, Figueiredo JC, Hu C, Molteni E, Canas L, Österdahl MF, Modat M, Sudre CH, Fox B, Hammers A, Wolf J, Capdevila J, Chan AT, David SP, Steves CJ, Ourselin S, Spector TD. Symptom prevalence, duration, and risk of hospital admission in individuals infected with SARS-CoV-2 during periods of omicron and delta variant dominance: a prospective observational study from the ZOE COVID Study. Lancet. 2022 Apr 23;399(10335):1618-1624. doi: 10.1016/S0140-6736(22)00327-0. Epub 2022 Apr 7. PMID: 35397851; PMCID: PMC8989396.

Tsang NNY, So HC, Ng KY, Cowling BJ, Leung GM, Ip DKM. Diagnostic performance of different sampling approaches for SARS-CoV-2 RT-PCR testing: a systematic review and meta-analysis. Lancet Infect Dis. 2021;21(9):1233-45.

As we have removed discussion of “Collider Bias” from the manuscript (see relevant reviewer comment and explanation below), the total number of references for the paper has increased by 3. Ultimately, we would like to keep the scope of the discussion focused towards the implications of our survey and so have avoided steering the paper into a larger review of paediatric screening and diagnostics.

**25) Supporting Information/"Appendix": Not sure if I see the utility of attaching these. STROBE is not referenced anywhere else that I noticed, so what is it supporting? The survey “appendix” seems a bit repetitive to the main text and is only referenced once (reference should be to SI, not Appendices).**

The STROBE checklist submitted was not intended to be included as part of the actual manuscript or publication, but rather to demonstrate fulfillment of the quality standards for our survey. We apologize for this confusion, and have removed it from our Supporting Information. We also now recognize that the actual survey itself, if included, should be labelled as a document within the Supporting Information as opposed to a document within an “Appendix Section”. To fit with PloS ONE submission guidelines, we are submitting the survey as a file in the Supporting Information section and have labelled it “S1 Child/Caregiver Survey”. As the exact wording of the survey is relevant to how reader’s may interpret the results, we feel an obligation to make access to it available. We do, of course, defer to the Editors on whether or not to include a link to the survey in the publication. Reference to the survey has been edited to appear as “see Supporting Information” instead of “see Appendix”, and appears in the manuscript as:

**Materials and Methods (Lines 135 to 136)**

Primary decision makers rated their willingness to accept a test on a 5-point Likert scale (e.g., very unlikely to very likely; (see *Supporting Information* ~~Appendix~~*)*.

**Minor comments and style suggestions (please take or leave):**

We greatly appreciate the helpful suggestions for improving the style and formatting of our document. We have addressed each of the following:

**26)** **Throughout – Ref numbers and Figure refs on the wrong side of the punctuation or with missing spaces between ref and text.**

All articles and figure/table references have been reformatted with the reference put after punctuation, without space unless the reference is to a specific element of a sentence.

**27)** **Title - Line 1: typo in SARS-CoV-2**

The title has been amended to read SARS-CoV-2 instead of SARs-CoV-2, appearing now as:

Nasopharyngeal Swabs vs. Saliva~~-based~~ Sampling for SAR*S*-CoV-2 Detection: A Cross-Sectional Survey of Acceptability for Caregivers and Children after Experiencing Both Methods

**28)** **Line 46: “prior” instead of “a priori”**

The original line in this instance read “Excluded were those with a priori identified inability to complete the consent, sampling, or survey process.” The term “a priori” in this context was meant to denote that patients were excluded when their their inability to participate was determined prior to attempting to complete consent, sampling or the survey; as opposed to being excluded if they had difficulty after already starting the process. For clarity, the sentence has been changed to say:

**Abstract (Lines 48 to 49)**

“Excluded were those identified prior to participation as having inability to	complete the consent, sampling, or survey process.”

**29)** **Line 62: “factors that influence sampling” instead of “factors sampling”**

We accept this modification, and the line is now:

**Abstract (Lines 62 to 63)**

*“Understanding factors that influence sampling acceptance will inform more	successful testing strategies.”*

**30) Line 66 and 68: parentheses instead of square brackets**

Square brackets around abbreviations have been replaced with parentheses. The relevant line of text is now:

**Introduction (Lines 67 to 68)**

*“Using nasopharyngeal swabs for SARS-CoV-2 gene detection via reverse	transcriptase	polymerase chain reaction (RT-PCR) testing is the current criterion	standard diagnostic test.”*

**31)** **Line 70: hyphen in “under-represented”**

The text now appears as:

**Introduction (Lines 76 to 77)**

*“While pediatric data is under-represented in this literature, the presumption has	been that saliva sampling would be particularly preferable for children.”*

**32)** **Line 72: delete “It has been shown that”**

This phrase was deleted.

**33)** **Line 95: “decision” instead of “decisions” – Just curious, when is assent appropriate?**

The word “decisions” was amended to “decision”.

Generally speaking, assent is appropriate in paediatric research when the child participant is not developmentally capable of providing true informed consent, but is still mature enough to provide affirmative agreement to participate. All participants in our study whose caregivers consented on their behalf were still asked for assent. To avoid any confusion, the sentence has been modified to read:

**Materials and Methods (Lines 105 to 107)**

*“Written informed consent was obtained from all participants or their primary	decision makers, and assent was obtained ~~when appropriate~~ from children whose	caregivers were consenting on their behalf.”*

**34)** **Line 110: Adolescents – what is exact age group?**

Adolescents in this context specifically refers to children 12 years old and older. We have now included this in the text:

**Material and Method (Lines 125 to 126)**

*“Adolescents (12 years old and older) could identify as being their own primary	medical decision makers.”*

**35) Line 113: “our own” instead of “a de novo”**

We have replaced “a de novo” as suggested, instead simply using the phrase “a survey”:

**Materials and Methods (Lines 127 to 129)**

*“As no validated tool to assess our primary outcome existed, we developed ~~a de~~	~~novo~~ a survey according to principles in the Association for Medical Education in	Europe (AMEE) guide to questionnaire development.”*

**36)** **Line 118: italicize e.g.**

All instances of “*e.g.*” throughout the manuscript have now been italicized.

**37) Line 119: Too much punctuation - “likely, see Appendix)” instead of “likely; (see Appendix)) Response:**

We have amended this line and the associated punctuation to read in a cleaner manner. It is now:

**Materials and Methods (Lines 135 to 136)**

***“****Primary decision makers rated their willingness to accept a test on a 5-point Likert	scale (“Very unlikely to Very likely”, see Supporting Information)."*

**38)** **Line 120: Square brackets look weird to me in paragraph text.**

The square brackets in question have been changed to parentheses. The line is now:

**Materials and Methods (Line 137)**

***“****Procedural pain was assessed using the validated Faces Pain Scale-Revised	(FPS-R).”*

**39) Line 121: “by asking yes or no” instead of “with direct binary”, “concluding” instead of “final”**

We have accepted this suggestion, and the text is now:

**Materials and Methods (Lines 137 to 142)**

*“Participants’ preference for sampling modality was assessed by asking them to	indicate which they would choose if a future test was required, and the desire for an	intranasal numbing agent was assessed with ~~direct binary questions~~ a yes or no	question. ~~In the final hypothetical scenario the primary decision maker had to~~	~~consider a potential trade-off of test comfort versus test sensitivity.~~ Additionally,	primary decision makers were asked to consider a potential trade-off of test	comfort versus test sensitivity by indicating their test preference in a hypothetical	scenario where saliva sampling was 10% less sensitive than a nasopharyngeal	swab.”*

**40)** **Line 129-31: A bit wordy… “were dichotomized to likely and not likely (including neutral).”**

We appreciate this suggestion, and the sentence is now:

**Materials and Methods (Lines 147 to 148)**

*“To facilitate data analysis, answers to the 5-point Likert were dichotomized~~. Likely~~	~~and very likely were coded as “likely”, whereas very unlikely, unlikely and neutral~~	~~answers were coded into “not likely”.~~ to likely and unlikely (including neutral	answers).”*

**41)** **Line 142: Wordy… “Convenience sample size was 50 participants”.**

We have taken this suggestion, and the sentence is now:

**Materials and Methods (Line 160)**

*“We determined a ~~pragmatic~~ convenience sample size of 50 participants. ~~in our~~	~~study’s protocol before beginning data collection.”~~*

**42) Table 1 – Needs cleaning up, too many ugly square brackets! Spaces after commas. No need for “+” or “[%]” or “, yes” – Remove extra space before “CEGEP”.**

We have have applied all of these recommendations to Table 1. It now appears as:

| **Table 1. Demographics** |  |
| --- | --- |
| Participants | 48 |
| Age, years (Median, IQR) | 10 (9,13) |
| Sex, female | 24 (50%) |
| Saliva sampling method |  |
| *Sponge* | 8 (17%) |
| *Expectoration* | 40 (83%) |
| Had an NP Swabs before this visit? | 23 (48%) |
| # NP Swabs for participants (Median, IQR) | 1 (1,3) |
| Primary decision maker had an NP Swabs? | 27 (57%) |
| # NP swabs for primary decision maker (Median, IQR) | 1 (0,2) |
| Symptoms | 18 (38%) |
| *Fever* | 5 (10%) |
| *Respiratory* | 2 (4%) |
| *Nasopharyngeal* | 9 (19%) |
| *Gastroenterologic* | 11 (23%) |
| *Non-specific* | 11 (23%) |
| Exposure to COVID contact | 31 (65%) |
| Primary decision maker education level |  |
| *Less than high school* | 1 (2%) |
| *High school* | 1 (2%) |
| *College/Trade school/CEGEP* | 15 (31%) |
| *University* | 31 (65%) |
| Abbreviations: IQR = interquartile range, NP = Nasopharyngeal | |

**43) Line 174 and 176: Space before “ 2”. Space before “ 3”.**

Spaces were added between “Fig.” and the figure numbers so that they appears as:

**Main Results (Lines 193 to 197)**

*“Fifty-eight percent of children rated their pain to be significant (4/10 or higher) on	the FPS-R during the nasopharyngeal swab, whereas no participant described	significant pain during saliva sampling.(Fig. 2) Ninety-six percent of children	reported 0/10 pain with saliva sampling. Ninety percent of children would prefer to	have saliva sampling in the future if they had to be tested again.(Fig. 3)”*

**44)** **Line 182: Period after “vs.”**

The correction was made so that a period now appears as part of “vs.”:

**Main Results (Lines 202 to 205)**

*“In this scenario, primary decision makers preferred a nasopharyngeal swab less	frequently in younger children (< 12 years) compared to older children (> 12 years)	(55% vs. 88% respectively).”*

**45) Line 182-4: Sounds like the parent wants the numbing spray for themselves – “prefer a nasal numbing spray be offered to their child” ?**

To make it clear that parents where asked to indicate if they would want a nasal numbing for their children with future COVID-19 nasopharyngeal swabs, the sentence was modified to:

**Main Results (Lines 207 to 209)**

*“55% of primary decision makers indicated they would want their child to receive a	nasal numbing spray prior to their ~~child having an NP~~ nasopharyngeal swab even	if it meant waiting 10 minutes for the spray to have an effect.”*

**46)** **Line 193: “acceptability” instead of “acceptably”**

This typo was corrected and the sentence is now:

**Discussion (Lines 216 to 217)**

*“Unlike saliva sampling, however, the acceptability of nasopharyngeal swabs was	different when the indication for testing changed.”*

**47) Line 195: “contact of” instead of “contact with”, comma after “case,”**

The suggested corrections were made so that the text is now:

**Discussion (Lines 217 to 219)**

*“Primary decision makers were less accepting of future nasopharyngeal swabs	when their child was an asymptomatic contact of ~~with~~ a COVID-positive case,	compared to when it was a requirement for returning to school.”*

**48)** **Line 203: “et al.” instead of “et. al.”**

The requested correction of “*et al.*” was made in the text.
**49)** **Line 212: “not testing is less obvious” instead of “not testing is not as obvious”**

Thank you for this wording suggestion. The sentence now reads:

**Discussion (Lines 237 to 240)**

*“Primary decision makers in our investigation may have been less accepting of a	nasopharyngeal swab in the context of asymptomatic contact with a positive	COVID case because the consequence of not testing is less ~~not as~~ obvious or	impactful, compared to the scenario where their child cannot return to school	without a test.”*

**50)** **Line 215: “testing is perceived as less valuable” instead of “the value of testing is not seen as high”, italicize e.g.**

Thank you as well for this wording recommendation. We have have modified the sentence to be:

**Discussion (Lines 240 to 243)**

*“Given that saliva ~~SB~~ sampling was highly accepted in all the proposed scenarios, it	may be that primary decision makers are more likely to accept this modality over a	nasopharyngeal swab when testing is perceived as less valuable ~~when the value of~~	~~testing is not seen as high~~ (e.g., voluntary asymptomatic screening, mild or atypical	COVID-19 symptoms).”*

**51) Line 219: “most effectively” instead of “best”**

We have edited this suggestion into the text:

**Discussion (Lines 245 to 248)**

*“Further study and delineation of this variability will help determine which	sampling options, in which clinical scenarios, most effectively ~~best~~ balance test	reliability with caregiver willingness to have children tested.”*

**52)** **Line 225: “likely varies in different” instead of “is likely different in differen**t”

We have incorporated this improved phrasing so that the text is now:

**Discussion (Lines 251 to 252)**

*“The actual difference in sensitivity between saliva sampling and nasopharyngeal	swabs is not definitively known at present, and likely varies in different ~~is likely~~	~~different in different~~ clinical scenarios.”*

**53)** **Line 226-7: italicize “et al.”**

*“et al.”* now appears appropriately italicized throughout the manuscript
**54)** **Line 227: “that sensitivity” instead of “that te sensitivity”**

This section of the text was changed to reflect an additional reference, and this wording was corrected along with other changes. See comment 56) below for the new text.

**55)** **Line 229: “ranged from 22-94%” instead of “to be as low as 21.9% and as high as 93.9%”**

This section of the text was changed to reflect the addition of another reference, and so this wording no longer appears. See comment 56) below for the new text.

**56) Line 229-30: “comparator” instead of “gold standard”**

This section of the text was changed to reflect the addition of another reference, and so this wording no longer appears. See below for the new text:

**Discussion (Lines 253 to 258)**

*“While initial meta-analyses have estimated sensitivity of saliva samples to be	similar to nasopharyngeal swabs,(3, 4), ~~a subsequent study by~~ Mestagh et al.(14)	found that the ~~te~~ sensitivity varied widely according to saliva collection technique	and viral load. ~~Using a similar outpatient setting and testing indications as for our~~	~~study, they found sensitivity in SB sampling to be as low as 21.9% and as high as~~	~~93.9% using the NP swab result as the gold standard.~~ Tan et al.(15) also found that	test performance varied significantly according to saliva collection and processing	technique, and suggested in their review that saliva assays may perform better	than nasopharyngeal swabs in optimal conditions."*

**57)** **Line 231: no hyphen in “ongoing”**

We have removed the hyphen from the word “ongoing”.
**58) Line 238: no hyphen in “inpatient”**

We have removed the hyphen from the word “inpatient”.

**59)** **Line 239: “understanding feasibility and acceptability of saliva-based screening” instead of** **“understanding acceptance”**

We have ultimately chosen to edit out the redundant part of this sentence, which removes the phrase “with the aim of understanding acceptance” completely. It now appears as:

**Discussion (Lines 269 to 271)**

*“Future studies should further elucidate how different populations respond to the	experience of COVID-19 testing in other settings, ~~with the aim of understanding~~	~~acceptance.~~ such as the inpatient setting or when patients are highly symptomatic.”*

**60)** **Line 241: “since” instead of “as”**

We incorporated this wording change into the sentence, which is also addressed below in the next comment, 61), from the reviewer.

**61)** **Line 242-3: “designed a survey to assess our primary outcome, based on best practices (6).”**

We incorporated this wording change into the sentence, which is now written as:

**Limitations (Lines 278 to 280)**

*“First, since ~~as~~ no validated survey to assess SARS-CoV-2 test acceptability exists in	the literature, ~~we had to develop a survey to assess our primary outcome; however,~~	~~our survey design was informed by best-evidence.~~ we designed a survey to assess	our primary outcome, based on best practices.(8)”*

**62)** **Line 247: Briefly explain what is a “collider”?**

We thank the reviewer for asking us for this clarification. While working on this definition, we re-examined our position, and now retract that a limitation of this study is Berkman’s bias (which is a specific form of collider bias). Instead, we believe there is a less concerning, more common issue of generalizability from our sample and have added text to describe this.

**Limitations (Lines 288-290)**

*“… this sample consists of those who went to assessment centers for testing and	then consented to be included in a research study, which may affect	generalizability.”*

Here we provide our reasoning for removing our Berkman’s bias / collider bias statements. Collider bias is a term from the field of causal inference, where there is a third variable which the predictor and the outcome variables point to. Berkman’s paradox is one specific example of a collider bias (see diagram).

Berkman’s bias is common in COVID-19 studies when data from assessment centers are used to examine the outcome of a test’s positivity.

Herbert, A., Griffith, G., Hemani, G. and Zuccolo, L. (2020), The spectre of Berkson's paradox: Collider bias in Covid-19 research. Significance, 17: 6-7. https://doi.org/10.1111/1740-9713.01413

In contrast, our studies outcome (comfort of test) does not have a causal pathway ‘back-door’ where the comfort level of test impacts likelihood to arrive at a testing center (see diagram, where the outcome only has arrows pointing towards it). Further, we have a repeated measures design, where patients got both tests.

For additional reading on this topic see this [popular science coverage of Berkman’s bias](https://slate.com/human-interest/2014/06/berksons-fallacy-why-are-handsome-men-such-jerks.html) and work by Miguel Herna (Prof of Biostatistics at Harvard).

**63)** **Line 248: “youth and their families” instead of just “youth”, “is valuable regardless” instead of “justifies the risk”**

We have incorporated this suggestion so that the sentence is now:

**Limitations (Lines 282 to 283)**

*“However, the benefit of obtaining the opinions of youth and families who	experienced testing with both modalities j~~ustifies the risk of~~ is valuable regardless of	selection bias.”*

**64)** **Line 249-51: Axe the last two sentences of this paragraph.**

As per the recommendation of the reviewer, we have removed the last two sentences of the original paragraph. No longer appearing in the text are the following lines:

*~~“In addition, families who voluntarily sought COVID-19 testing would have~~	~~important perspectives regarding future testing. Finally, we enrolled a~~	~~convenience sample of 50 participants; a larger sample size might have increased~~	~~the power to detect statistical differences. “~~*

**65) Line 256: “varied” instead of “is more prone to vary”**

The sentence has been modified to read:

**Conclusion (Lines 291 to 292)**

*“Acceptance of nasopharyngeal swabs, however, ~~is more prone to vary~~* *varied based	on testing rationale.”*

**66) Line 257: “SARS” instead of “Mers”**

We apologize for this typographical error, and all reference to “Mers” has been changed to “SARS”

**67)** **Line 259: “supercede” is mis-spelled**

“supercede” has been changed to the preferred spelling, “supersede”
**68)** **Line 261: “comparable” instead of “non-inferior or equivalent”, “SARS” instead of “Mers”**

The text has been edited to reflect this more efficient wording suggestion, and now appears as:

**Conclusion (Lines 294 to 296)**

*“Patient and family preferences should be considered by public health policy	makers, especially when testing modalities are ~~non-inferior or equivalent~~	comparable.”*

**69)** **Line 262: “ideal application for each modality” instead of “ideal use for the differing modalities”.**

The reviewer suggestion is much appreciated, and the wording now appears as:

**Conclusion (Lines 296 to 297**

*“Further study of SARS-CoV-2 sampling accuracy and acceptability will help	determine the ideal ~~use for the differing modalities~~ application for each modality.”*

**Line 269-74: Add cédille to “François”**

The reviewers acknowledgement of the cultural and personal significance attached to the use of the cédille in this author’s name is particularly appreciated. All instances of François’ name now have the cédille added.
